# ICTV Virus Taxonomy Profile: *Parvoviridae*

**DOI:** 10.1099/jgv.0.001212

**Published:** 2019-01-23

**Authors:** Susan F. Cotmore, Mavis Agbandje-McKenna, Marta Canuti, John A. Chiorini, Anna-Maria Eis-Hubinger, Joseph Hughes, Mario Mietzsch, Sejal Modha, Mylène Ogliastro, Judit J. Pénzes, David J. Pintel, Jianming Qiu, Maria Soderlund-Venermo, Peter Tattersall, Peter Tijssen, ICTV Report Consortium

**Affiliations:** ^1^​ Department of Laboratory Medicine, Yale University School of Medicine, New Haven, CT 06520- 8035, USA; ^2^​ Department of Biochemistry and Molecular Biology, University of Florida, Gainesville, FL 32610, USA; ^3^​ Department of Biology, Memorial University of Newfoundland, St John’s, NL A1B3X9, Canada; ^4^​ National Institute of Dental and Craniofacial Research, National Institutes of Health, Bethesda, MD 20892, USA; ^5^​ Institute of Virology, University of Bonn Medical Centre, Bonn D-53105, Germany; ^6^​ MRC - University of Glasgow Centre for Virus Research, Glasgow G61 1QH, UK; ^7^​ INRA-Université de Montpellier, 34095 Montpellier Cedex 5, France; ^8^​ Department of Molecular Microbiology and Immunology, University of Missouri, Columbia, MO 65211, USA; ^9^​ Department of Microbiology, Molecular Genetics and Immunology, University of Kansas Medical Center, Kansas City, KS 66160, USA; ^10^​ Department of Virology, University of Helsinki, FIN-00014 University of Helsinki, Finland; ^11^​ Department of Genetics, Yale University School of Medicine, New Haven, CT 06520-8035, USA; ^12^​ Centre de Recherche de Microbiologie et Biotechnologie, INRS-Institut Armand-Frappier Laval, QC H7V 1B7, Canada

**Keywords:** *Parvoviridae*, *Parvovirinae*, *Densovirinae*, taxonomy, ICTV Report

## Abstract

Members of the family *Parvoviridae* are small, resilient, non-enveloped viruses with linear, single-stranded DNA genomes of 4–6 kb. Viruses in two subfamilies, the *Parvovirinae* and *Densovirinae*, are distinguished primarily by their respective ability to infect vertebrates (including humans) versus invertebrates. Being genetically limited, most parvoviruses require actively dividing host cells and are host and/or tissue specific. Some cause diseases, which range from subclinical to lethal. A few require co-infection with helper viruses from other families. This is a summary of the International Committee on Taxonomy of Viruses (ICTV) Report on the *Parvoviridae*, which is available at www.ictv.global/report/parvoviridae.

## Virion

**Fig. 1.**
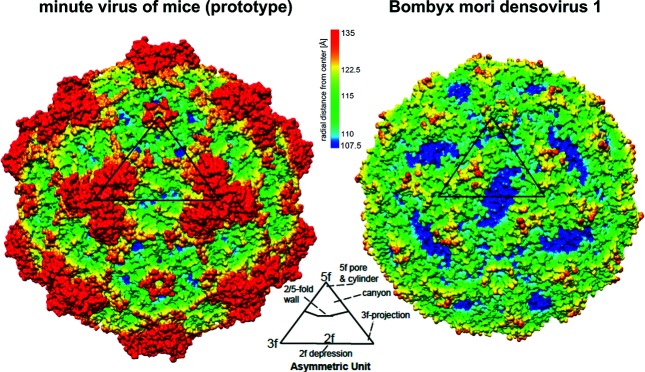
Virion morphology. Three-dimensional X-ray diffraction structures of exemplar parvoviruses at 3.1–3.4 Å resolution, obtained using PDBs 1MVM and 3P0S. Colour depicts distance from the virus centre; triangles outline one of 60 icosahedral units showing the 2-, 3- and 5-fold axes of symmetry.

Parvovirus virions are small, rugged, non-enveloped protein particles with T=1 icosahedral symmetry (Table 1 and Fig. 1). A single coat protein sequence is expressed as a nested set of virion proteins (VP) with a common C-terminal domain that forms the virion shell. VP1 N-termini may have phospholipase A2 (PLA_2_) activity [1, 2].

**Table 1.** Characteristics of the family *Parvoviridae*

Typical member:	human parvovirus B19-J35 G1 (AY386330), species *Primate erythroparvovirus 1*, genus *Erythroparvovirus*, subfamily *Parvovirinae*
Virion	Small, non-enveloped, T=1 icosahedra, 23–28 nm in diameter
Genome	Linear, single-stranded DNA of 4–6 kb with short terminal hairpins
Replication	Rolling hairpin replication, a linear adaptation of rolling circle replication. Dynamic hairpin telomeres prime complementary strand and duplex strand-displacement synthesis; high mutation and recombination rates
Translation	Capped mRNAs; co-linear ORFs accessed by alternative splicing, non-consensus initiation or leaky scanning
Host range	*Parvovirinae*: mammals, birds, reptiles. *Densovirinae*: insects, crustacea, echinoderms
Taxonomy	Two subfamilies, *Parvovirinae* and *Densovirinae*; 13 genera, >75 species

## Genome

**Fig. 2.**
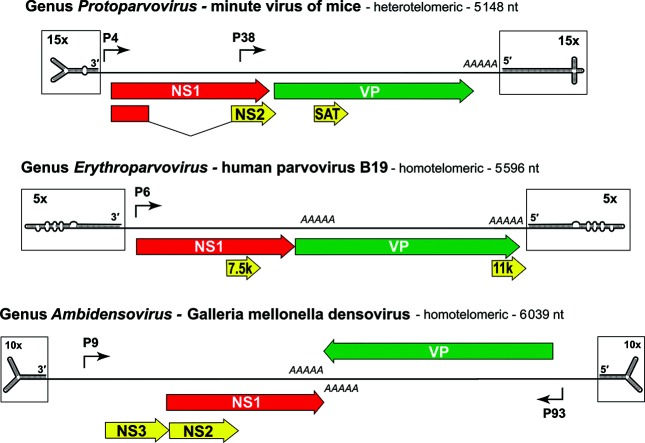
Parvovirus genome organization shows genus-specific variations. Terminal hairpins are magnified relative to the coding region to show predicted secondary structures. ORFs are indicated by arrowed boxes. Angled arrows indicate transcriptional promoters and *AAAAA* indicates polyadenylation sites.

Viruses package a single copy of a linear ssDNA molecule of 4–6 kb, which contains a long coding region bracketed by short (120–600 nt) dynamic hairpin termini that mediate DNA replication (Fig. 2). Packaged strands can be of negative or both senses. Two gene cassettes encode a replication initiator protein (NS1 or Rep) and a virion protein (VP), plus a few small genus-specific auxiliary proteins. Many parvoviruses are highly specialized for infecting particular host cells. Since host restrictions may be relaxed when cells undergo oncogenic transformation, viruses in some species, such as *Rodent protoparvovirus 1*, may be preferentially oncolytic [3]. In contrast, adeno-associated viruses co-opt helper viruses, such as adenoviruses or herpesviruses, to support their productive replication; the simplicity, durability, broad tissue specificity and lack of toxicity makes these viruses useful gene transfer vehicles for research and clinical studies [4].

## Replication

Parvoviruses bind glycosylated cell surface molecules and are internalized by receptor-mediated endocytosis. Virions are metastable [1]; in endosomes some undergo a conformational shift, exposing VP1 PLA_2_ domains required for lipid bilayer penetration. Intact virions enter the nucleus, where their genomic 3′-hairpin primes complementary strand synthesis by a host replication fork. This creates a duplex transcription template, allowing RNA polymerase II transcription to initiate gene expression (Fig. 2)

DNA replication proceeds via a ‘rolling hairpin’ mechanism, which relies on sequential unfolding and refolding of the hairpin termini. Unidirectional strand displacement synthesis generates continuous duplex intermediates, from which progeny single strands are excised by the endonuclease activity of NS1. Progeny genomes are packaged into preassembled viral particles by the NS1 helicase, via a portal at one of the icosahedral 5-fold axes. Progeny virions may be rapidly exported from living cells, or accumulate in the nucleus until liberated by cell lysis. Many viruses cause mild disease, whereas others, such as canine parvovirus (species *Carnivore protoparvovirus 1*) and most members of the subfamily *Densovirinae*, are highly pathogenic [2, 5].

## Taxonomy

Members of the subfamily *Parvovirinae* infect vertebrates (mammals, birds and reptiles). This subfamily includes eight genera; *Bocaparvovirus*, *Dependoparvovirus*, *Erythroparvovirus*, *Protoparvovirus*, *Tetraparvovirus*, *Amdoparvovirus*, *Aveparvovirus* and *Copiparvovirus*, containing >55 species. Viruses that infect humans are present in seven species from the first five genera in this list. Members of subfamily *Densovirinae* infect invertebrates (insects, crustaceans and echinoderms). This subfamily contains one unassigned species and five genera; *Ambidensovirus*, *Brevidensovirus*, *Hepandensovirus*, *Iteradensovirus* and *Penstyldensovirus*, comprising >20 species.

## Resources

Full ICTV Report on the family *Parvoviridae*: www.ictv.global/report/parvoviridae.
